# Chronic Kidney Disease: A Public Health Problem That Needs a Public Health Action Plan

**Published:** 2006-03-15

**Authors:** Anton C Schoolwerth, Michael M Engelgau, Kathy H Rufo, Frank Vinicor, Thomas H Hostetter, Dolph Chianchiano, William M McClellan, David G Warnock

**Affiliations:** Section of Hypertension/Nephrology, Dartmouth–Hitchcock Medical Center; Division of Diabetes Translation, Centers for Disease Control and Prevention, Atlanta, Ga; Division of Diabetes Translation, Centers for Disease Control and Prevention, Atlanta, Ga; Division of Diabetes Translation, Centers for Disease Control and Prevention, Atlanta, Ga; National Kidney Disease Education Program, National Institute of Diabetes and Digestive and Kidney Diseases, National Institutes of Health, Bethesda, Md; National Kidney Foundation, New York, NY; Department of Epidemiology, Rollins School of Public Health, Emory University, Atlanta, Ga; Department of Medicine, University of Alabama at Birmingham, Birmingham, Ala

## Abstract

For a health problem or condition to be considered a public health issue, four criteria must be met: 1) the health condition must place a large burden on society, a burden that is getting larger despite existing control efforts; 2) the burden must be distributed unfairly (i.e., certain segments of the population are unequally affected); 3) there must be evidence that *upstream* preventive strategies could substantially reduce the burden of the condition; and 4) such preventive strategies are not yet in place. Chronic kidney disease meets these criteria for a public health issue. Therefore, as a complement to clinical approaches to controlling it, a broad and coordinated public health approach will be necessary to meet the burgeoning health, economic, and societal challenges of chronic kidney disease.

## Chronic Kidney Disease: A Public Health Problem?

Health problems or conditions become public health issues when four criteria are met (1,2). First, the disease burden is high (i.e., it affects many people, has increased recently, and will likely increase in the future). This burden is experienced in terms of mortality and morbidity, quality of life, and cost and is perceived as a threat by the public; that is, there is a sense of fear that the disease is out of control. Second, the problem is distributed unfairly (i.e., it does not affect all people the same but affects minorities and disadvantaged individuals to a greater extent). Third, there is evidence that *upstream* preventive strategies — strategies that target economic, political, and environmental factors that affect a population's health — could substantially reduce the burden of the condition; and fourth, evidence shows that such preventive strategies are not yet in place.

Chronic kidney disease (CKD) meets these criteria for consideration as a public health issue. For the first criterion, there are now more than 385,000 people in the United States with end-stage renal disease (ESRD) (3), the form of CKD in which life can be sustained only by dialysis or transplantation. Milder forms of CKD that do not yet require renal replacement therapy are even more common than ESRD. The total number of Americans living with CKD is now estimated to be 19.2 million, representing 11% of the adult U.S. population; the 0.22% of the population estimated to have ESRD (4) comes from this large group of individuals with early CKD.

The burden of CKD is growing. During the past three decades, the incidence and prevalence of ESRD have risen progressively. For example, annual new cases of ESRD increased from approximately 14,500 in 1978 to 100,359 in 2002; during the same period, the number of individuals on dialysis and with kidney transplants increased from 42,000 to 431,000 (3,5). Estimates for 1993 to 1995 were that 2% of white men, 1.7% of white women, 5.5% of black men, and 6.3% of black women would develop ESRD during their lifetime (6). Five years later, however, these estimates had increased to 2.5% of white men, 1.8% of white women, 7.3% of black men, and 7.8% of black women (7). Projections to the year 2010 estimate an annual 4.1% increase in incident ESRD cases, although recent data from the U.S. Renal Data System (USRDS) indicate that the rate of increase is lessening (5,8). By 2030, it is estimated that the annual number of people with new onset of ESRD will exceed 450,000, and those receiving dialysis or who have had kidney transplants will exceed 2 million (9).

CKD causes premature morbidity and mortality and lowers quality of life; it is also expensive. Yearly death rates of ESRD patients are approximately 20%. CKD patients have a risk of cardiovascular disease (CVD) that is 10 to 30 times that of people without kidney disease (10). Recently, data from several large, diverse populations have shown that progressive decreases in the glomerular filtration rate (GFR) were associated with increased risks of death, cardiovascular events, and hospitalization; these risks were fewer than those reported in ESRD patients (11,12). Deaths caused by CKD were estimated at 71,000 in 2000 and are expected to increase to 352,000 in 2030 (9). Recently, using data from death certificates, the Centers for Disease Control and Prevention (CDC) listed kidney disease as the ninth leading cause of death in the United States (13). However, this statistic underestimates the burden of kidney disease because it does not reflect the high rates of comorbidity in the CKD population. Indeed, CKD patients have a greater likelihood of dying from comorbidities of kidney disease than of progressing to ESRD (14,15). In addition to reducing lifespan, CKD substantially reduces quality of life, and yet it is often not recognized as a serious health problem in the United States.

Treating ESRD imposes a large economic burden on patients, the health care system, and society. Although people with kidney failure represent less than 1% of the Medicare population (individuals with kidney failure, regardless of age, are eligible for Medicare funding), their care consumes 6.4% of the health care expenditures by the Centers for Medicare and Medicaid Services. In 2001, total expenditures (Medicare plus private payers) exceeded $22 billion, of which approximately two thirds was provided by Medicare (5). In addition, recent data from a large HMO and from the USRDS indicate that the total health care resources used for CKD patients are 1.6 to 2.4 times (or more) those resources used by the ESRD population (16). The fact that one in nine Americans is estimated to have CKD and another 20 million are at risk for developing it has resulted in fear that the disease is out of control.

CKD also meets the second criterion of a public health issue: it disproportionately affects racial and ethnic minorities, among whom worse outcomes and higher costs of treatment are common. African Americans and American Indians are at especially increased risk (17). International data suggest that CKD is a worldwide public health problem (17). Age alone is a key predictor of CKD, and 11% of people in the United States aged 65 years or older (without diabetes or hypertension) have moderately to severely decreased kidney function (4). Currently diabetes is the most common cause of kidney failure, now accounting for nearly one half of new cases of ESRD, and by 2006 it is expected to surpass all other causes of new cases combined (i.e., hypertension, glomerulonephritis, and others) (9).

The third and fourth criteria are also satisfied by CKD: it is feasible to act on the condition at the community and public health levels. Despite the tremendous burden of CKD, there is good news — we have the requisite knowledge to prevent or at least delay its onset, its progression, and the comorbidities that accompany it. Upstream preventive strategies are not yet in place but if implemented effectively could reduce the burden of CKD.

## Potential for Prevention of CKD

Fortunately, the large burden of CKD does not appear to be inevitable; there are many reasons to believe it can be reduced substantially. A key will be the early identification of individuals who are at risk. There is evidence that earlier stages of CKD can be detected and treated and that adverse outcomes of CKD can be prevented or delayed (17).

Clinical diagnosis of CKD has become simplified. The most sensitive test for early CKD is urine albumin. The earliest stage of low-grade albumin leakage into urine is called *microalbuminuria.* Current recommendations call for annual urine testing of people with diabetes (18-20). Although recommendations for testing for other risk groups have not been established, testing for proteinuria, at least with simple dipstick methods, has been calculated to be cost effective in people with hypertension (21). Also, the GFR (the major kidney function test) can be reasonably estimated from serum creatinine using an equation validated in a large number of subjects with CKD in combination with the variables of age, sex, and race (18). Thus, a determination of serum creatinine level and a spot urine sample for albumin–creatinine ratio are sufficient to detect CKD.

Current preventive care practices include maintaining stringent control of blood pressure to a target of 130/80 mm Hg, using angiotensin-converting enzyme inhibitors (ACEIs) and angiotensin II receptor blockers (ARBs) in both diabetic and nondiabetic nephropathies, maintaining careful glycemic control in individuals with diabetes, and following a low-protein diet (21). Additional reports indicate that treating dyslipidemia, losing weight, quitting smoking, and managing anemia may also help to delay progression of early CKD; however, some of these results must be tempered by the fact that relatively few patients were enrolled in these studies (21,22).

The benefits of treating early kidney disease may extend beyond the kidney itself. Indeed, a recent publication indicated that in the general population, the presence of albuminuria (a key indicator of kidney disease) predicted both cardiovascular and noncardiovascular mortality (23). It has been suggested that in many cases, microalbuminuria is simply the renal manifestation of a generalized abnormality of vascular function (24). A recent report showed that using fosinopril (an ACEI) to treat individuals who were identified from screening as having microalbuminuria led to a reduction in both albuminuria and cardiovascular events (25), the latter the major cause of death in patients with CKD (10). However, these results were obtained from a relatively small sample size with lack of statistical significance of the cardiovascular events, so confirmatory results from a larger trial are needed.

Several studies have demonstrated the potential for preventing or delaying the initial onset of diabetic kidney disease by treating patients who have diabetes with ACEIs. ACEIs prevent the development of microalbuminuria. In the early stages of diabetes, patients may have heightened renal function, which manifests itself as a high GFR, sometimes called *hyperfiltration*. Such a state may precede the development of microalbuminuria in diabetes (21).

Unfortunately, many patients with CKD still receive suboptimal care (26-28). The disease is both underdiagnosed and undertreated. The reasons for this suboptimal care are likely complex, but people at risk because of diabetes or hypertension are often unaware that CKD can be caused by these conditions. In addition, screening with quantitative urinary albumin measurements is inadequately performed in patients with diabetes. Also, the usual clinical index of kidney function, the serum creatinine concentration, is often poorly interpreted by clinicians.

## A Public Health Problem That Needs a Public Health Approach

CKD is not being detected early enough to initiate treatment regimens and reduce death and disability (17). In addition, many interventions are being delivered too late to improve population-based outcomes. Finally, most individuals with CKD are unaware that they have this disorder (17,29). Thus, the issue of CKD extends beyond a clinical problem addressed only by health care providers to a major public health issue requiring multilevel efforts. Initiatives should be undertaken to make health care providers and the general population more aware of the seriousness of CKD, its risk factors, and opportunities for screening. People identified with CKD should be provided appropriate educational materials to explain the treatment regimens and the benefits of undertaking therapy. We must work with health care delivery organizations to ensure access to high-quality care, and we must provide data and information to health care policy makers so that their decisions will effectively address CKD.

The USRDS collects, analyzes, and distributes information on ESRD patients (3,5). Currently, however, there is no data surveillance system for tracking patients with CKD in stages before dialysis or transplantation, unless they are aged 65 years or older and covered by Medicare (and thus can be tracked by the Centers for Medicare and Medicaid Services). CDC has a national surveillance system in place for diabetes (available from www.cdc.gov/diabetes), and there are national surveillance efforts with the Behavioral Risk Factor Surveillance System (BRFSS), National Health and Nutrition Examination Survey (NHANES), National Health Interview Survey (NHIS), and National Hospital Discharge Survey (NHDS), among others, to assess cardiovascular disease. However, data are scant for CKD. Clearly, to get a better understanding of the nature and extent of the CKD burden and to inform policy decisions, national surveillance data on this disorder need to be made available.

Additional public health efforts to address CKD are sorely needed, but some important first steps have been taken. These include publication of clinical practice guidelines for CKD by the National Kidney Foundation–Kidney Disease Outcomes Quality Initiative (NKF–K/DOQI) (17), a meeting of stakeholders to assess priorities (30), and the establishment of the National Kidney Disease Education Program (NKDEP) (31). Sponsored by the National Institute of Diabetes and Digestive and Kidney Diseases, NKDEP was created to reduce morbidity and mortality from kidney disease and its complications. Through public education and system-level initiatives such as improving the reporting by clinical laboratories of kidney function, NKDEP aims to raise awareness that kidney disease is serious, that it is important to test people at risk, and that treatment is available to prevent entirely or slow the progression of the disease (31).

Several additional elements are needed to address CKD effectively. A comprehensive effort will require patient education, professional education, and the involvement of payers (Medicare, Medicaid, and the health insurance industry). In addition, the involvement or cooperation of business, the community, and government will be required; national, state, and local initiatives will all be needed. More research efforts will be needed to measure and track the CKD burden, identify populations at risk, and target program efforts.

## Conclusion

The burden of CKD, in terms of human suffering and economic costs, is exploding as we move through the early years of the 21st century, making it a major public health issue. We know how to prevent or delay the onset of CKD and to limit its progression. Unfortunately, the extent to which we have applied this knowledge, which can effectively reduce the burden of CKD, is disappointing. A comprehensive public health approach will be needed to effectively address this major health problem.
